# Case Report: Biallelic *BRCA1* pathogenic alterations in a Fanconi Anemia patient and clinical implications of variant location

**DOI:** 10.3389/fonc.2025.1572310

**Published:** 2025-05-30

**Authors:** Colin C. Young, Ashley Lahr, Caroline Nestor, Ashley Kaminski, Marcy E. Richardson, Georgianne L. Arnold

**Affiliations:** ^1^ Ambry Genetics, 1 Enterprise, Aliso Viejo, CA, United States; ^2^ University of Pittsburgh Medical Center, Pittsburgh, PA, United States

**Keywords:** oncology, BRCA1, Fanconi anaemia, splicing, case report

## Abstract

Pathogenic alterations in *BRCA1* are associated with autosomal dominant breast and ovarian cancer and autosomal recessive Fanconi Anemia Subtype S (FA-S). FA-S accounts for <1% of all reported cases of FA with only ten patients identified in the literature to-date. Here we describe an eleventh FA-S proband with severe microcephaly, growth failure, duodenal stenosis, hyperpigmented macules, dysmorphic features, and abnormal chromosomal breakage, consistent with other FA-S patients. Two pathogenic *BRCA1* variants (c.191G>A, p.C64Y and c.3991C>T, p.Q1331*) were identified in *trans*. At four years old, this patient has not been diagnosed with cancer or bone marrow failure, which are hallmark features in other subtypes of FA. Like a majority of the literature-reported FA-S patients, this patient harbors a truncating variant in *BRCA1* exon 11. This exon undergoes alternative splicing resulting in a protein with partial BRCA1 activity. The retained activity may be enough to rescue an otherwise lethal phenotype explaining the viability of FA-S patients. This retained functional activity may also modify clinical cancer risks and treatment implications for heterozygous carriers of exon 11 truncating variants. This work further characterizes the features of FA-S patients and discusses a molecular hypothesis for the rarity and viability of individuals with this condition.

## Introduction

Pathogenic alterations in *BRCA1* are linked to a high-penetrance, autosomal dominant cancer predisposition syndrome (MONDO: 0700268, OMIM: 604370) (1, 2). Women who are heterozygous carriers of pathogenic *BRCA1* alterations have significantly increased lifetime risks of developing breast and ovarian cancer and both male and female heterozygous carriers are at increased risk for pancreatic cancer (3). The identification of patients who carry these alterations has significant implications on cancer screening recommendations, surgical management that includes prophylactic surgery, family planning, and familial testing to reduce cancer risk and decrease mortality.

More rarely discussed, however, is the risk of Fanconi Anemia Subtype S (FA-S; MONDO:0054748, OMIM:617883) which is an autosomal recessive disorder observed in patients with biallelic pathogenic alterations in *BRCA1* (4). FA-S is an extremely rare condition and accounts for fewer than 1% of all FA cases (4). Thus far, only ten cases are described in the literature (5–11). The presentation of FA-S includes pre- and postnatal growth failure, microcephaly, developmental delay, hyperpigmented macules, and dysmorphic features. Other abnormalities include hip dysplasia, duodenal stenosis, and limb defects. None of the literature patients reported bone marrow failure, which is a common feature in other FA subtypes (12).

A chromosomal breakage test using either diepoxybutane (DEB) or mitomycin-C (MMC) can provide a molecular diagnosis of FA and eight of ten FA-S patients had abnormal chromosomal breakage (4). Eight of nine of these patients had at least one nonsense or frameshifting alteration in *BRCA1* exon 11 (coding exon 9), and one had a frameshifting alteration in exon 10. *BRCA1* knock-out mice are embryonic lethal (13), leading to questions about the mechanisms which lead to viability of FA-S patients in whom two loss-of-function alleles are identified (14).

Here we report an eleventh FA-S proband with biallelic pathogenic variants in *BRCA1*. The proband has physical and developmental characteristics consistent with the other FA-S patients reported in the literature to date. Molecular confirmation of FA-S was confirmed by chromosomal breakage analysis and phase confirmation was determined by parental testing. As with the majority of other FA-S patients, a nonsense alteration in exon 11 was identified as one of the alterations.

## Patient and methods

### Clinical testing and ethics

Peripheral blood samples and clinical histories were collected from the proband and both parents as part of diagnostic testing at Ambry Genetics and GeneDx. Chromosomal breakage analysis was performed on patient peripheral blood lymphocytes by the Comprehensive Center for Fanconi Anemia at Dana Farber Cancer Institute. An informed consent form was obtained by the patient’s parent/guardian for this publication.

### Variant classification

Variant classification was performed using modified ACMG/AMP clinical classification guidelines as recommended by the ClinGen ENIGMA BRCA1 and BRCA2 Variant Curation Expert Panel (VCEP) (https://cspec.genome.network/cspec/ui/svi/doc/GN092).

## Results

### Patient phenotype

The female proband was born prematurely at 34 weeks to non-consanguineous parents. Prenatally, the proband was small for gestational age, had microcephaly (<3^rd^ percentile), and intrauterine growth restriction. Prenatal karyotype, chromosome SNP microarray, and FISH were normal. The proband’s parents were of African American and Caucasian descent, were developmentally normal, and four maternal half-siblings were reported to be healthy.

After birth, the proband was identified to have two duodenal webs which required surgical repair. Ophthalmological exam showed unilateral retinal coloboma. A brain MRI noted simplified gyral pattern, colpocpehaly, and a partially dysplastic corpus callosum, which were expected to result in significant impacts on neurodevelopment. A cardiac exam showed a patent foramen ovale. The proband displayed developmental dysplasia of the left hip. Additional examinations noted the patient had bitemporal narrowing of the forehead, up-slanting palpebral fissures, a bulbous nose, bilateral clinodactyly, single palmar crease, and bilateral toe overlap.

Both whole exome sequencing and multi-gene panel testing were performed on the proband and identified a variant of uncertain significance in *BMP4* (c.351G>A, p.V117V) and two pathogenic alterations in *BRCA1*: c.191G>A, p.C64Y (maternally inherited) and c.3991C>T, p.Q1331* (paternally inherited). No other variants were identified in FA-associated genes. The family cancer history, while is limited, includes a maternal grandmother with breast cancer at 38. Both are classified by multiple submitters as pathogenic in ClinVar.

Chromosomal breakage analysis determined the patient was positive for FA. Treatment of patient blood lymphocytes with MMC resulted in an abnormal result of 2.96 aberrations/cell (FA negative and FA positive controls were 0.2 and 4.84 aberrations/cell, respectively). 48% of patient cells had radial formations (negative and positive controls were 4% and 76%, respectively). Treatment with DEB showed similar results with 3.26 aberrations/cell (versus 0 and 4.40 aberrations/cell for positive and negative controls, respectively) and 30% of patient cells with radial formations (versus 0% and 64% for positive and negative controls, respectively. No spontaneous breakage was observed in the patient sample.

By age 2, the patient had 10 café-au-lait macules larger than 5 mm, delayed developmental milestones, and no other features of neurofibromatosis. The patient had persistent microcephaly (<2 SD) and was small in stature. The complete blood count showed mild macrocytosis, but normal leukocyte and platelet levels, supporting no evidence of bone marrow failure. As of writing, the patient is four years of age, has not developed cancer, and is living, but has been lost to follow up.

## Discussion

The clinical characteristics of the proband detailed here closely match the clinical characteristics identified in the other FA-S patients, as recently reviewed (5, 15). This phenotype includes pre- and post-natal growth failure (10/11), microcephaly (11/11), developmental delay (9/11), and hyperpigmented spots (10/11) (5–11). Other common features include limb defects (8/11), microphthalmia (5/11), and structural and skeletal defects such as duodenal stenosis and hip dysplasia (8/11). In contrast to other FA subtypes, none of the 11 FA-S patients had bone marrow failure, including those who have reached adulthood. Due to the rarity of reported FA-S patients, cancer risk cannot be calculated, however six of the ten had been diagnosed with cancer by age 30 and included breast and ovarian in the older patients and leukemia, neuroblastoma, and a brain tumor with the earliest onset at age 13 months. While FA-S appears to result in early on-set of a spectrum of cancers, it does not appear to be as penetrant as FA-D1 (caused by biallelic loss of *BRCA2*), for example, in which malignancy has been reported to be observed in up to 97% of patients before age 6 (16).

It is expected that a human embryo with two fully penetrant, pathogenic *BRCA1* variants would not be viable based on three observations: 1) the extreme dearth of FA-S cases; 2) the absence of reports of individuals homozygous or compound heterozygous for the two high-frequency founder pathogenic variants in *BRCA1* [c.68_69delAG, p.E23Vfs*17: maximum allele frequency 0.4% in Ashkenazi Jewish-gnomAD v4.0) and c.5266dupC, p.Q1756Pfs*74: maximum allele frequency 0.1% in Ashkenazi Jewish-gnomAD v4.0] (17); and 3) the observation that *brca1* knock-out mice are early embryonic lethal (13). One hypothesis for the viability of FA-S patients is the partial retained function of at least one of the pair of pathogenic alterations. The recurrent observation of FA-S patients with different pathogenic alterations in exon 11 supports this hypothesis as this exon is excluded in well-studied, alternative, in-frame splicing isoforms that whose protein products retain at least partial BRCA1 protein function (6, 7, 10, 15, 18, 19). The most predominant natural, in-frame events include 1) the splicing-out of exons 9 and 10 (r.548_670del); 2) the splicing-out of exons 9-11 (r.548_4096del); and 3) the splicing-out of part of exon 11 (r.788_4096del). The splicing-out of solely exon 11 (r.670_4096del) is observed but is less prevalent.

**Figure 1:**
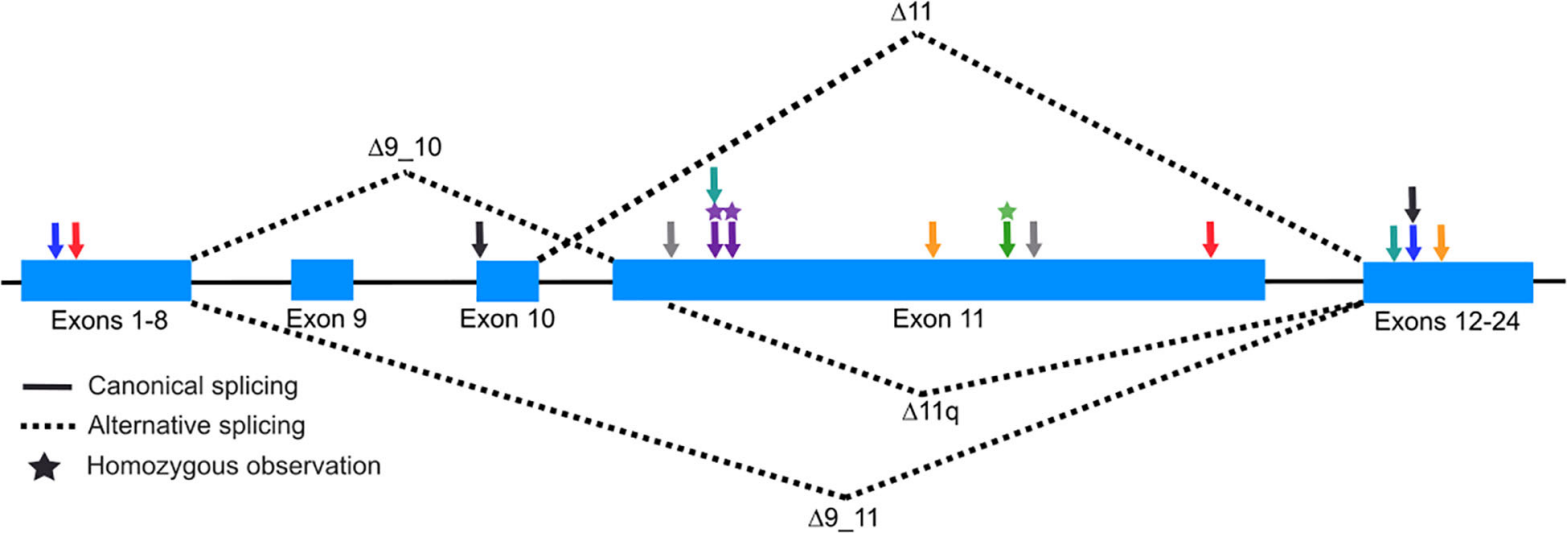
Illustration of *BRCA1* alternative splicing isoforms. Schematic (not to scale) of *BRCA1* highlighting exons 9 through 11 (coding exons 7 through 9). Solid lines between exons represent canonical splicing and dashed lines represent splicing isoforms which remove exons 9 and 10 (Δ9_10), exons 9-11 (Δ9_11), a majority of exon 11 (Δ11q), and exon 11 (Δ11). Arrows represent the approximate location of *BRCA1* variants identified in FA-S patients (Black, Sawyer et al.; Blue, Kuepp et al.; Yellow, Domcheck et al.; Green, Freire et al; Purple, Seo et al.; Gray, Chirita-Emandi et al.; Teal, Borlin et al.; Red, this report).

BRCA1 protein functional studies showed that, despite removing ~60% of the of the protein, loss of exon 11, which is not part of a known functional domain, retains partial homology-directed DNA repair activity (19, 20). In FA-S patients, the natural splicing-out of exon 11 removes the truncating alterations originating here in these patients, which in-turn could lead to the recovery of partial function of BRCA1 proteins originating from this allele. Thus, the recurrence of exon 11 loss of function alterations in FA-S patients is not by chance, but rather variant location is a mechanistically important factor for viability (**Figure 1**, **Table 1**).

**Table 1:** *BRCA1* germline alterations and protein impacts identified in FA-S patients.

Study	Allele 1 Nucleotide Change	Protein Impact	Exon	Allele 2 Nucleotide Change	Protein Impact	Exon
Sawyer et al. (9)	c.594_597delTGTG	p.S198Rfs*35	10	c.5095C>T	p.R1699W	18
Keupp et al. (8)	c.181T>G	p.C61G	5	c.5096G>A	p.R1699Q**^†^**	18
Domcheck et al. (6)	c.2457delC	p.D821Ifs*25	11	c.5207T>C	p.V1736A	18
Freire et al. (7)	c.2709T>A	p.C903*	11	c.2709T>A	p.C903*	11
Seo et al. (2 patients) (10)	c.1115G>A	p.W372*	11	c.1115G>A	p.W372*	11
Seo et al. (2 patients) (10)	c.1292T>G	p.L431*	11	c.1292T>G	p.L431*	11
Chirita-Emandi et al. (5)	c.843_846delCTCA	p.S282Yfs*15	11	c.2933dupA	p.Y978*	11
Borlin et al. (11)	c.1116G>A	p.W372*	11	c.5017_5019delCAC	p.H1673del	17
This study	c.191G>A	p.C64Y	5	c.3991C>T	p.Q1331*	11

^†^ - Functionally hypomorphic, moderate risk alteration (22).

The splicing, functional, and FA-S clinical and genetic data suggest that *BRCA1* exon 11 nonsense and frameshifting alterations are definitionally hypomorphic and not complete loss-of-function alleles. The residual function of the alternative splicing isoforms could have an impact on the clinical risk associated with the development of cancers and the clinical management for carriers of these alterations in heterozygous individuals. Analyses of cancer risk conferred by pathogenic variants in *BRCA1* exon 11 show a reduction in breast cancer risk, but a relative increase in ovarian cancer risk compared to the other regions of the protein (21). Additionally, both *in vitro* and *in vivo* murine functional studies have shown that alternative splicing of exon 11 leads to an increase in resistance to PARP inhibitors and cisplatin due to the expression and retained function of proteins arising from these isoforms (19). Interestingly, cells from the FA-S proband described in this work displayed intermediate sensitivity to DEB and MMC relative to the positive control, which may also be attributable to the retained function of proteins arising from alternative exon 11 splicing. This retained function may also be a partial explanation for why the phenotypic characteristics of FA-S patients are distinct from other FA subtypes, such as the absence of bone marrow failure (15).

Baseline ACMG/AMP variant interpretation guidelines suggest that loss-of-function variants can be assigned the PVS1 code when a variant results in a null allele, however, exon 11 frameshift and nonsense alterations do not strictly meet these criteria. While available evidence suggests that nonsense and frameshift alterations in *BRCA1* exon 11 confer an increased risk for cancer, the risk profile and clinical management guidelines for heterozygous individuals may be attenuated relative to other complete loss-of-function alterations in *BRCA1.* Further study of the genotype-phenotype relationship for heterozygous carriers of *BRCA1* exon 11 nonsense and frameshifting variants is warranted to help inform clinical care.

While this work has been mostly focused on alternative splicing as a mode for FA-S patient viability due to partial protein function, missense variants, such as those identified in the FA-S proband in Keupp et al. are another mode for partial protein function (8). The proband in that work had similar clinical features as other FA-S patients but was surprisingly negative by chromosomal breakage analysis. A proposed explanation for the normal chromosomal breakage results is the known reduced risk (relative to other truncating *BRCA1* variants) of *BRCA1* c.5096G>A, p.R1699Q which maintains partial BRCA1 function (22, 23).

## Conclusions

Here we identify an eleventh FA-S patient with biallelic *BRCA1* pathogenic alterations confirmed in *trans* through parental genetic testing. Phenotypic presentation in the patient was consistent with the other FA-S probands in the published literature including pre- and postnatal growth failure, developmental delay, severe microcephaly, dysmorphic features, and café-au-lait spots. Chromosomal breakage analysis was positive using both DEB and MMC. At four years of age, the patient is living and has not been diagnosed with cancer or bone marrow failure.

Most FA-S patients have a truncating variant in exon 11 and here we provide a hypothesis as-to how alternative splicing of this exon supports that this concentration of variants is not by chance, rather a mechanism for non-lethality of FA-S patients conferred by retained partial protein function. The partially retained function may also have implications for the cancer risk and clinical management of individuals heterozygous truncating variants originating in exon 11.

FA-S patients are at increased risk of developing cancers, including breast and ovarian, at an early age. The exact risk profile and spectrum of cancers associated with FA-S patients will require more time and identification of additional probands to fully characterize, but screening and management as with other FA patients is likely warranted.

## Data Availability

The datasets presented in this study can be found in online repositories. The names of the repository/repositories and accession number(s) can be found below: https://www.ncbi.nlm.nih.gov/, VCV000054400.69 https://www.ncbi.nlm.nih.gov/, VCV000037558.11.
